# Strategies to Mitigate Biofouling of Nanocomposite Polymer-Based Membranes in Contact with Blood

**DOI:** 10.3390/membranes13090762

**Published:** 2023-08-28

**Authors:** Dominika Wójtowicz, Ewa Stodolak-Zych

**Affiliations:** 1 Department of Biomaterials and Composites, Faculty of Materials Science and Ceramics, AGH University of Krakow, Al. Mickiewicza 30, 30-059 Krakow, Poland; stodolak@agh.edu.pl; 2 Clinical Department of Anaesthesiology and Intensive Care, University Hospital in Krakow, ul. Jakubowskiego 2, 30-688 Krakow, Poland

**Keywords:** polymer membrane, biofouling, hemofilter, CRRT, nanoparticles, nanocomposites

## Abstract

An extracorporeal blood purification method called continuous renal replacement therapy uses a porous hollow-fiber polymeric membrane that is exposed to prolonged contact with blood. In that condition, like with any other submerged filtration membrane, the hemofilter loses its properties over time and use resulting in a rapid decline in flux. The most significant reason for this loss is the formation of a biofilm. Protein, blood cells and bacterial cells attach to the membrane surface in complex and fluctuating processes. Anticoagulation allows for longer patency of vascular access and a longer lifespan of the membrane. Other preventive measures include the modification of the membrane itself. In this article, we focused on the role of nanoadditives in the mitigation of biofouling. Nanoparticles such as graphene, carbon nanotubes, and silica effectively change surface properties towards more hydrophilic, affect pore size and distribution, decrease protein adsorption and damage bacteria cells. As a result, membranes modified with nanoparticles show better flow parameters, longer lifespan and increased hemocompatibility.

## 1. Introduction

Membranes have a broad range of applications in industry and medicine. One common application is the use of membrane processes such as diffusion and ultrafiltration in a blood purification technique called CRRT (continuous renal replacement therapy) in patients with AKI (acute kidney injury). A porous hollow-fiber polymeric membrane is used to remove from patients’ blood inorganic solutes, organic uremic toxins (Table 1), excess water, and in some cases, septic shock, and the overproduction of cytokines. A distinguished feature of this therapy is prolonged blood-membrane contact for up to 72 h. After that, the whole set used for CRRT needs to be changed.

In that condition, like with any other submerged filtration membrane, the hemofilter loses its properties over time and use which results in a rapid decline in flux and makes it impossible to accurately assess the effectiveness of applied therapy. The most significant reason for this loss is the formation of a so-called second membrane when a biofilm layer is formed within minutes of contact with aqueous solutions [1]. The main factor of fouling in low-pressure membranes such as MF and UF is organic matter composed of proteins, polysaccharides and bacteria cells suspended in feed [2]. The issue of biofouling on blood-contacting membranes is more complex since various blood cells are deposited on the membrane and may activate feedback loops acting as additional promoting factors.

The harmful effects of biofilm in healthcare-related situations include not only the insufficient regulation of serum levels of solutes due to permeation flux decline and selectivity failure but also other adverse effects on the patients. In order to prevent biofilm formation, the membrane’s properties should be better tailored, e.g., by modifications during development. It can be accomplished by adding nanomaterials to polymer solution prior to casting film. In this review, we focused on an approach based on nanoadditives: carbon nanotubes (CNTs), graphene and nanosilica and the application of their antimicrobial properties to limit biofilm formation and enhance the existing properties of membranes.

**Figure 1:**
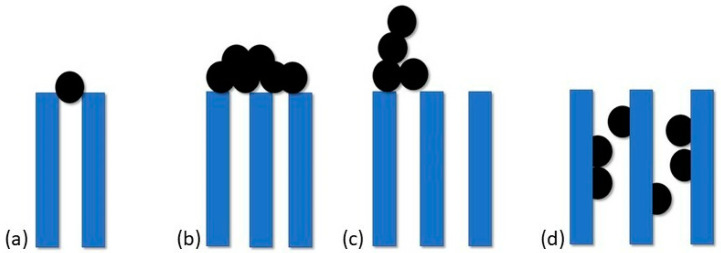
Schematic representation of fouling mechanism: (**a**) complete blocking (**b**) cake filtration (**c**) intermediate blocking (**d**) standard blocking.

Processes behind the decline in flux are under thorough research [3,4,5,6]. According to filtration laws, we can distinguish four types of pore-blocking mechanisms as depicted in Figure 1: standard blocking, complete blocking, intermediate blocking and cake filtration. Wang et al. [7] during their study proved that cake filtration is the last step of a membrane fouling process, but it is not a dominant explanation for flux decline. Prior to this phenomenon, other pore-blocking mechanisms occur and in the case of UF membranes with properties similar to membranes used in CRRT, first appear intermediate blocking followed by standard blocking and the last step is cake filtration (although there are times when these processes happen simultaneously).

Commercial hemofilters are described by set clearance coefficients for the most common solutes such as creatinine, urea, and phosphate. These tests are carried out with solutions without plasma proteins thus they do not provide us with information about the behavior of the membrane during the actual therapy when the biofilm layer creates an additional barrier for uremic toxins removal [1]. In his research, Kimura [8] proved that biofouling is irreversible by comparing the used membrane with the one used but cleaned chemically and physically with a sponge. There was a clear correlation between hydrophilic biopolymer concentrations in a feed and fouling rates, gradually leading to a loss in membrane efficiency.

In a study on the development of bacterial layers during cross-flow filtration, Eshed et al. demonstrated a ~75% decrease in permeability after 48 h, of which a 22% decrease occurred within the first hour [6]. Additionally, the membrane lifespan expectancy was shortened.

The aim of the work is to address the challenges associated with the use of membrane processes in CRRT, especially ones related to the decline in the effectiveness of therapy and put it in the clinical aspect. In particular, we intend to better understand factors contributing to biofilm formation on the porous hollow-fiber polymeric membrane, which negatively impacts the filtration process and the removal of solutes and toxins from the blood. To prevent this phenomenon, nanomaterials such as carbon nanotubes, graphene, and nanosilica are proposed as additives to the dope polymer solution to tailor membrane permeability, selectivity and properties. The included brief description of methods for analyzing biofilm on filtration membranes is necessary in order to recognize and investigate its role in membrane longevity and permeability decline.

## 2. Protein Adsorption onto the Membrane Surface

The clinical performance of biomedical devices is limited by the contact of protein and cells with the surface. In particular, HD (hemodialysis) membranes are subject to dynamic interactions between plasma proteins and the membrane surface: a process involving constant adsorption and desorption as a result of hydrophobic interactions and hydrogen bonds, electrostatic, ionic and Van der Waals forces [9].

In a process called the Vroman effect plasma proteins reversibly adhere to the surface and commonly get replaced in time by different types of proteins. Attached particles create a fouling layer with a complex and constantly evolving composition, so it is impossible to determine the structure of a given surface in detail [10,11]. The thickness varies between 2 and 10 nm, while the concentration of proteins on the surface can be 1000-fold greater than the concentration of proteins in plasma. It limits the effectiveness of diffusion and convection processes and reduces solute removal, especially the clearance of medium and large molecules [9,12].

The first proteins to adsorb onto the membrane surface are the ones most abundant in blood. Among them, albumin and fibrinogen are considered to be molecules that initiate layer formation. Their content in the fouling layer gradually decreases as they get replaced by coagulation factors from the contact pathway including factor XII, high molecular weight kininogen, prekallikrein and factor XI. This binding onto the membrane is competitive and makes proteins undergo conformational changes which uncovers access locations for blood cells or proteins thus further promoting adsorption [9,12].

In protein adsorption, the surface chemistry and physical properties of biomaterials play a significant role. It appears that the Vroman effect is independent of flow and is most evident on negatively charged hydrophilic surfaces [12]. Moreover, a hydrophilic surface is less prone to protein adsorption showing anti-fouling properties [9].

## 3. Bacteria Adhesion

**Figure 2:**
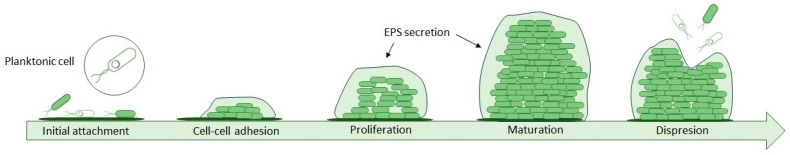
Five steps of the biofilm development process.

Proteins and chemical compounds suspended in the feed attach to the surface of the membrane changing the properties of the surface to favor the adhesion of bacteria. This formation of a conditioning film is the first phase of biofilm formation, a complex process that can be described in five steps (Figure 2). On such adjusted surface, cells are easily deposited. Once they settle, they start to organize into microcolonies and secrete an extracellular polymeric substance (EPS) which provides structural integrity by irreversibly bonding cells and offering them protection. It consists of a mix of polysaccharides, proteins, D-amino acids, fatty acids and a variety of nucleic acids and accounts for 80–90% of biofilm mass. Over time biofilm grows thicker and newly deposited layers vary in bacteria species, composition and oxidation level. The last step, when bacteria cells located in the top layer get dispersed back into the surrounding fluid, is called sloughing [13,14,15].

Biofilms are multi-species. The microbiological component of the biofilm consists of Gram-positive bacteria, Gram-negative bacteria and fungi. For medical devices, representative pathogenic Gram-positive species include: *Staphylococcus aureus*, *Staphylococcus epidermidis*, *Enterococcus faecalis* and *Streptococcus viridans* and Gram-negative ones including *Pseudomonas aeruginosa*, *Escherichia coli*, *Klebsiella pneumoniae* and *Proteus mirabilis* [14,16]. Studies on strains of pathogenic bacteria like Staphylococcus (*S. aureus*, *S. epidermidis*) and Streptococcus showed structures expressed on bacteria membranes called microbial surface components recognizing adhesive matrix molecules (MSCRAMMs) which are responsible for the ligand-receptor binding with plasma proteins, platelets or other cell of a given tissues [17].

The feed is the main source of bacteria and the rate of biofouling in the cross-flow filtration does not change whether the dragged bacteria are dead or viable [6]. However, the contamination may come from medical professionals during placement procedures or from the patient’s skin [16]. The type of bacteria also depends on the drugs administered through the catheter, e.g., during the infusion of catecholamines Gram-negative bacteria are more often isolated [18].

Several factors influence the rate of cell attachment, including the number and type of cells suspended in the fluid, the flow rate through the device, and the physicochemical properties of the surface [16]. Studies have shown that the formation of a biofilm begins rapidly, after about 1 min of immersion of the surface and because bacteria have a negative surface charge, especially in the early phases of the cell cycle, this process occurs faster on positively charged surfaces. Therefore, the first phase of adhesion depends mainly on hydrodynamic and physiochemical processes like electrostatic forces, van der Waals forces and acid-base interaction based on Lewis theory [15,19].

The second phase is irreversible and can take several hours depending on the species. An important role played by biological processes and changes in bacteria metabolism is associated mainly with different gene expression and the production of novel proteins. For example, in *Pseudomonas fluorescens* a new ABC transporter and a secretion of proteins are required for irreversible attachment to occur [15,20]. In addition, the same species show different properties, physiology and gene expression depending on whether they are planktonic or forming biofilm [21]. In the human environment, bacteria can incorporate host components such as immunoglobulins or platelets and fibrin into the biofilm matrix [22]. The composition of the biofilm changes dynamically over time: the pioneer bacteria first to adhere may be displaced by subsequent species or disappear over time, leaving behind a rich surface ready to accept succeeding colonizers.

## 4. Interactions with Blood Cells

It is hard to describe blood cell-membrane dynamic interaction in chronological order because many of these steps occur simultaneously and promote each other in a feedback loop. Platelets show a tendency to adhere to biomaterials which causes their activation and degranulation.

RGD peptide is an amino acid sequence (arginine-glycine-aspartate) mediating the attachment of numerous cell types to the surface of biomaterials. Integrins are transmembrane receptors responsible for cell adhesion, which recognize RGD sequence in ECM proteins such as fibronectin, vitronectin, fibrinogen or osteopontin and bind with them initiating the aggregation process [23]. An example of integrin expressed on a platelet membrane is very late antigen 5 (VLA-5) and GP IIb/IIIa, which binds RGD sequences of fibrinogen and fibronectin. Under static and dynamic conditions RGD is a crucial initiator of platelet deposition and the research confirms a direct correlation of fibrinogen adsorption with consequent platelet adherence onto artificial surfaces [24,25].

Activated platelets change their shape and release α-granules filled with fibrinogen, β-thromboglobulin, thrombospondin, vWF and fibronectin, substances that are procoagulant in nature and further stimulate thrombus formation [24]. Various pathways are likely to contribute to platelet activation, some of which are more relevant in the time of high complement and leukocyte activation and others under different health conditions.

Another cause of clotting is a bacteria-platelet interaction where binding takes place thanks to released ESP. Also, during an infection, a thrombus may form as a secondary effect of accompanying systemic platelet activation and DIC (disseminated intravascular coagulation). Some processes may even lead to the internalization of bacteria by platelets. The first description of this phenomenon comes from studies on *S. aureus* stimulated by ADP [17].

Surface-induced thrombosis occurs without a coagulation cascade in the absence of thrombin. On most polymer or metal surfaces where fibrinogen is readily adsorbed fibrin formation is spontaneous. This process requires a specific orientation of fibrinogen molecules: on the hydrophilic surfaces it takes a globular form, whereas on the hydrophobic surfaces, large fibers [26]. As proteins bind to a surface, platelet adhesion is promoted, which mediates further blood clotting.

**Figure 3:**
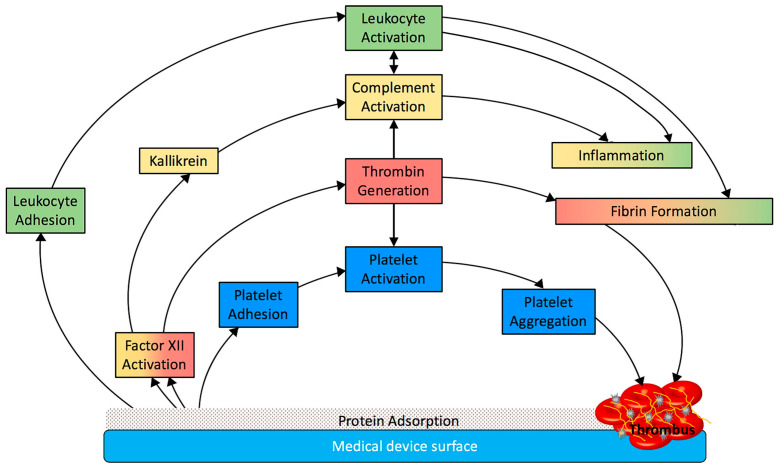
Dynamic interaction between protein and surface of biomaterials. Reprinted from Acta Biomaterialia 94, Jaffer, I.H.; Weitz, J.I. The Blood Compatibility Challenge. Part 1: Blood-Contacting Medical Devices: The Scope of the Problem; Pages No 2–10 [12], Copyright 2019 with permission from Elsevier.

These processes (summarized in Figure 3) are responsible for the thrombogenic properties of medical devices and further modification of the surface is needed to eliminate each one as the potential cause of biomaterial-related thrombosis.

## 5. Methods of Anticoagulation

Blood-contacting devices are prone to protein fouling that initiates a coagulation cascade and results in thrombus formation. In the case of HD, constant filtration of blood makes membranes even more susceptible to pore-blocking and may cause abrupt and complete clotting of the hemofilter. Excessive thrombus formation is controlled by the use of anticoagulation. It allows for longer patency of vascular access, but also a longer lifespan of the membrane.

The key to proper anticoagulation is to maintain a balance between thrombus formation and excessive bleeding in patients which may lead to further adverse events like hemorrhagic stroke and internal bleeding. Strategies include the use of heparin, both unfractionated and low molecular weight, regional citrate anticoagulation and novel membranes coated with heparin (e.g., oXiris) [27]. In some patients with initial coagulation disorder, there is a possibility to carry out CRRT without any anticoagulants. Every method has its indication and contraindication and should be adjusted to the patients’ general condition and doctors’ experience.

Unfractionated heparin (UFH) is made up of heparin molecules of different sizes between 5 and 30 kDa. It works by inhibiting factors IIa (thrombin) and Xa of the coagulation cascade which stops thrombus formation. During treatment, it is critical to monitor activated partial thromboplastin time (APTT) closely and obtain recommended values between 35 and 45 s. The APTT is a good predictor of filter clotting and hemorrhage in patients: studies have shown that UFH prolongs filter life proportionally to the APTT but not to the given dose of anticoagulant [28]. Heparin plasma half-life can extend up to 3 h with kidney injury which causes shifting in dosage and may cause unpredictable heparin blood levels further increasing the risk of hemorrhage. The incidence of bleeding events ranges from 10 to 50%, with mortality as high as 15% [29].

Citrate anticoagulation is a safe and effective alternative to heparin, it prolongs hemofilter patency and reduces bleeding complications (notably less bleeding and less blood transfusion in comparison with heparin) in critically ill patients [30]. Calcium ions are a coagulation factor IV that works in the last stage of the intrinsic and extrinsic coagulation cascades. Citrate binds and chelates free ionized calcium forming citrate–calcium complex, interrupting coagulation and thrombus formation.

Calcium is lost as the citrate–calcium complex via dialysis and filtration because it has a molecular weight of approximately 300 Da and can pass easily through the membrane. For that reason, the blood level of calcium ions must be restored before purified blood re-enters the circulatory system to ensure physiological systemic coagulation. Circuit’s and patient’s ionized calcium levels are measured frequently to adjust the dosage and guarantee efficient anticoagulation. The side effects are mainly connected to citrate accumulation and include metabolic alkalosis, metabolic acidosis, hypo- or hypercalcemia, hypernatremia and hypomagnesemia [27].

## 6. Methods for Analyzing Biofilm on Membranes

The levels of planktonic cells do not correlate with the scale of biofilm formation [20]. This is a reason why standard tests such as bacterial culture do not reflect the true extent of the biofilm and its biodiversity. Accumulation of ESP makes this analysis even more difficult by binding bacteria with each other. In addition, during the maturation stage, the biofilm grows thicker, which means that lower-lying species may also not be identified. There are a few methods that can confirm only the presence of biofilm and some, like flow cytometry, can give an exact number of detected bacteria, dead and viable, in one milliliter of the studied solution. To gather a full range of data we need to use a variety of methods, but we can select a technique dedicated to measuring exactly the requested biofilm parameters.

### 6.1. Flow Cytometry

Flow cytometry (FCM) is a non-destructive method that allows for the detection of any bacteria cell suspended in a fluid, no matter if in feed or permeate. During the test cells flow through a cytometer arranged in a single line while the laser beam is directed at them. The detector behind the cell detects changes in scattered light which gives characteristic parameters of studied substances. It can also be used to assess the stadium of biofilm formation and recognize if bacterial cells are sloughing from the ultrafiltrate membrane or if they are multiplying on the surface. The results are presented as an absolute number of bacteria cells, while the negative numbers represent bacteria removal. This method was repeatedly used to detect bacteria in drinking water [31].

Flow cytometry is slowly being replaced by PCR and DNA detection methods, which give more precise information. They also offer the possibility of using staining methods that can differentiate between dead and viable bacteria, e.g., by using a PMA (phosphomolybdic acid) stain that can penetrate only through torn cell membranes, bond to DNA and block it from replication during PCR [32].

### 6.2. X-ray Photoelectron Spectroscopy

X-ray photoelectron spectroscopy (XPS) is a destructive method of imaging samples of biofilm layer 2–5 nm deep. Results are represented as a histogram of energy levels characteristic of a given element and its chemical state, allowing the identification of the type of polysaccharide and protein that make up the EPS. The chemical characterization of the surface allows for the comparison of sterile and fouled membrane surfaces to demonstrate the development of a biofilm over time [33,34]. In a study on biofilm maturation on steel coupons it was shown that whilst the content of Fe and Cr decreased, the main components became C, O and N which indicates increasing biofilm accumulation. The surface film contained ~70% C after 28 days of exposure, whereas Fe and Cr were undetectable, indicating that the biofilm became thick enough to exceed the XPS detection limit [35]. As another application, XPS can be used to determine if the surface chemical composition has changed after biofilm removal [34]. In the same study after cleaning the surface, XPS showed a lower content of Fe and a higher content of Cr which may be the result of extensive micropitting caused by bacteria and EPS on the steel coupon [35].

### 6.3. Time of Flight SIMS

Secondary ion mass spectrometry (SIMS) is an imaging method that uses energy to induce the emission of characteristic particles of the surface. Released particles like ions or atoms are analyzed by a mass spectrometer, which allows to chemically characterize a surface, even of complex materials. Time-of-flight SIMS (ToF-SIMS) modification allows the collection of information on small metabolites, lipids and peptides with great precision and sensitivity. Commercial ToF-SIMS instruments allow for image acquisition with a spatial resolution of 400 nm and a mass resolution of 10,000 thanks to the delayed extraction of secondary ions [36,37]. This method provides more spectral information than XPS and can inform about fouling with other substances than organic matter, e.g., organosilicon [33].

### 6.4. Scanning Electron Microscopy

SEM (scanning electron microscopy) is used for imaging topography of a sample surface and in the case of biofilm detection it can show occupied area. It offers high-resolution visualization but lacks the perception of depth which creates distortion by imaging biofilm as individual cells on the surface. Unfortunately, it requires a dehydration process and further preparation prior to testing, which may cause breakage of the sample and the destruction of EPS [38,39,40].

Many researchers used SEM microscopy in their studies to demonstrate the advancement of biofilm formation and porosity of modified membranes [6,34,41]. However, one study compared different SEM techniques and features in capturing the image of *Streptococcus mutans* biofilms. Between SEM, SEM and ruthenium red (SEM-RR), and variable pressure scanning electron microscopy (VPSEM) (without the need to dehydrate a sample) the last one offered the best picture of the *S. mutans* biofilm morphology. Compared to SEM, the procedure for VPSEM is far less time-consuming, and no hazardous chemicals are required as in dehydration [40].

### 6.5. 16S rRNA Sequencing

One of the most precise methods is DNA profiling using primers specific for 16S rRNA which can be found in almost all bacteria. It is a gene sequencing method that targets 16S ribosomal subunit. It provides accurate information about microbes inhabiting the biomaterial and makes it possible to distinguish the species even between closely related bacterial taxa [42].

## 7. Characteristics of Membranes for CRRT

A polymeric hollow-fiber membrane is the most prevalent choice in the contemporary hemofilter production. It is characterized by high separation area, high permeability, high selectivity and excellent mass–transfer properties [43]. Hollow fibers have advantages over flat sheet membranes that ensure consistency of the purification process. They show better mechanical strength, larger pore areas with uniform size dispersion and low cost of production [44,45]. Membrane properties are also affected by the topology, the shape of the pores and the general porosity [46].

The surface topography may present antagonistic features that promote or mitigate adhesion and biofilm formation. As a consequence, the materials selected for hemodialysis membranes require deep consideration and precise testing of their biocompatibility. The first hemodialysis membranes were made with cellulose acetate but due to small pore size, complement activation and other side effects in patients, they are not commonly used. Novel polymers can withstand higher transmembrane pressure and do not ignite the inflammation response in patients. Among them are polysulfone (PSf), polyethersulfone (PES), polymethylmethacrylate (PMMA), ethylene vinyl alcohol (EVOH) and polyacrylonitrile (PAN). The overwhelming majority, as much as 93%, are derived from the polyarylsulfone family [44]. PSf membranes are preferred because of their chemical inertness and mechanical strength. They also show high thermal stability and can endure all sterilization techniques [47].

**Figure 4:**
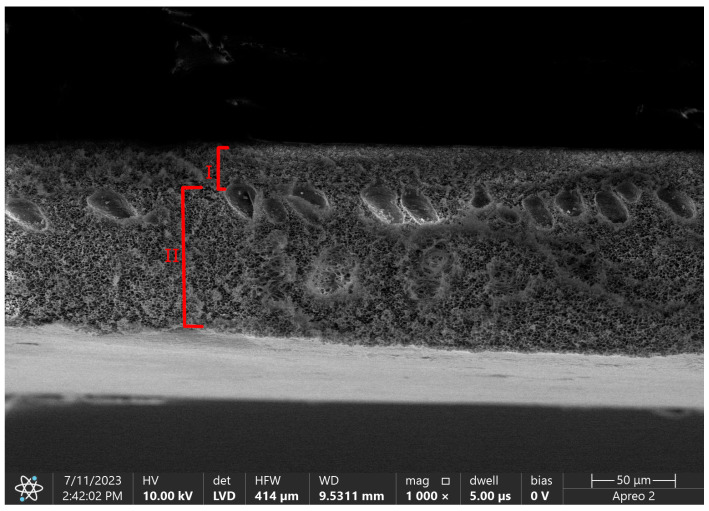
SEM micrograph of flat sheet pure polysulfone membrane with marked layers. I—skin layer, II—support layer. Own photo.

Using the phase inversion technique we are able to obtain asymmetric membranes with complicated porous structures divided into a skin layer and a support layer [43]. A skin layer, also called an active layer, is a blood-contacting side. Its thickness is a determinant of diffusion efficiency, with an inverse relationship between these two parameters [46]. The size of pores in this layer is also an eliminating factor for the size of removed molecules. The role of a support layer is to provide mechanical strength for the layer above, but also for the whole hollow-fiber structure that needs to withstand high-pressure differences during CRRT therapy. Figure 4 shows the difference in porosity between those layers in pure polysulfone flat sheet membrane.

One of the most crucial surface properties affecting biofilm formation is hydrophilicity. Hydrophilic surfaces show reduced protein adsorption and less nonspecific bacteria adhesion [44,48]. Additionally, due to reducing hemofilter performance and a decline in flux, protein adsorption on the membrane also affects its hemocompatibility. Upon the adsorption of coagulation factors onto the artificial surface, the proteins undergo conformational changes or denaturation, which promotes thrombus formation [1].

Another important factor is surface roughness. Greater roughness means a bigger surface area, which results in more sites of adhesion [19]. The roughness of the surface enhances its inherent wettability properties: increasing the surface area of hydrophobic materials makes it even more hydrophobic [49]. In one study, the contact angle was increased from 99.3° to 151.6° by increasing the roughness of polytetrafluoroethylene (PTFE) membranes, which showed a dual-reversible transition of wettability: upon alcohol prewetting or drying, the film could reversibly switch between superhydrophobicity and superhydrophilicity [50].

The adherence of bacteria and blood cells to polymeric biomaterials depends on surface chemistry and morphology. Other factors, such as plasma proteins, platelets, and fluid pH should also affect bacteria adherence [9]. Although over the years hemocompatibility aspects were improved, not so many advances were made in the field of fouling prevention. Due to its irreversible nature, the main emphasis should be put on methods mitigating biofilm formation.

## 8. Modifications of Polymer-Based Membranes

**Figure 5:**
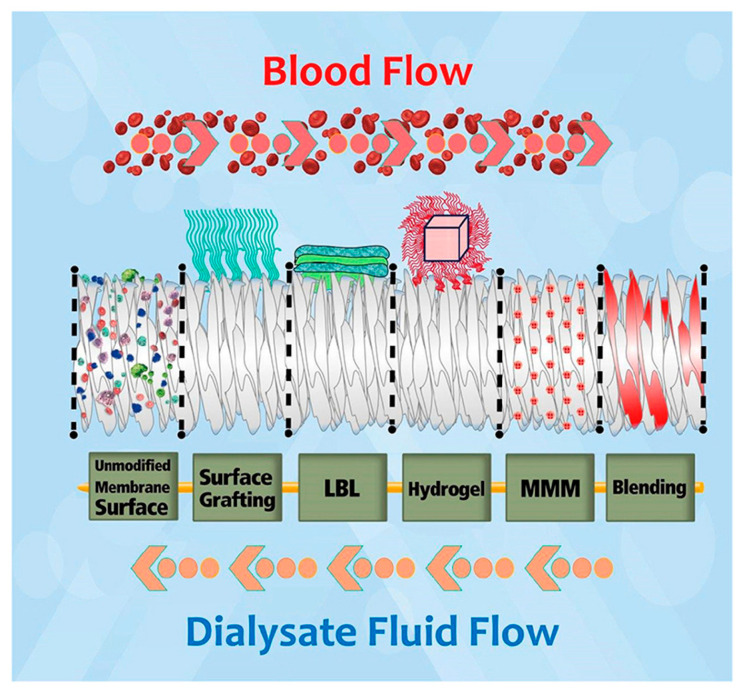
Modification techniques for polymeric hemodialysis membranes: chemical immobilization of functional groups (surface grafting), layer by layer chemical attachment of species (LBL), covalent attachment of super-hydrophilic hydrogel, mixed matrix membrane (MMM) and base polymer modification (blending). Reprinted from Materials Chemistry and Physics 248, Mollahosseini, A.; Abdelrasoul, A.; Shoker, A. A Critical Review of Recent Advances in Hemodialysis Membranes Hemocompatibility and Guidelines for Future Development [44]. Copyright 2020 with permission from Elsevier.

Numerous modifications have been attempted to improve the biocompatibility of hemodialysis membranes and to reduce biofouling. The variety of methods include polymeric blending, surface chemical modification, coating, grafting surfaces with heparin and hydrogel surface modification (Figure 5). Some of these adjustments are aimed at preventing bacteria adherence while others protect the membrane from excessive clotting. In this article, we wanted to focus on the composite materials and the role of nanoparticles such as graphene, carbon nanotubes and silica in the mitigation of biofouling in light of the conditions in which HD membranes are intended to function, e.g., prolonged contact with blood, excessive coagulation, variety of adhering proteins and different bacterial species. Such membranes must also show high biocompatibility for safe use in humans.

### 8.1. Graphene Modification Possibilities

The antibacterial properties of graphene and its derivatives like graphene oxide (GO) and reduced graphene oxide (RGO) involve the combination of three mechanisms: physical damage of cell membrane, oxidative stress that is independent of reactive oxygen species (ROS) and wrapping and trapping. The last one aims only at cells suspended in solutions depriving them of access to nutrients [51,52].

The study conducted by Song et al. shows that not-immobilized GO nanopowder with its extremely sharp edges is causing cell membrane rupture and subsequent necrosis. Moreover, the effect of GO on bacteria cells is considered to be dose-depended: in small concentrations (10 mg/L) it can increase biofilm formation because protein efflux from dead cells can be used as a source of nutrients and building materials for remaining cells, whereas in high dosage (80–160 mg/L) it inhibits further growth [41].

Graphene-based coatings show antimicrobial properties, which depend on the covered surface area and increase with every additional film layer. The overall effect is a 74% reduction in biomass, which is a little less effective in comparison with graphene suspended in solution (80%). This may be explained by the fact that only two out of three possible mechanisms are able to occur, excluding wrapping and trapping [51]. Graphene nanocoating on titanium surface shows a reduction in adhesion in Candida species, Gram-positive and Gram-negative bacteria and changes in biofilm morphology making it thin and fragmented [53].

Graphene oxide-based membranes (GOM) demonstrate properties that allow for more effective HD, especially in terms of better solute transport, reduction or discontinuation of anticoagulation therapy and improved fluid control. They show high sorption capacity, expandable dimension and better pore selectivity for ions and small molecules due to the possibility of many chemical modifications that allow the passage of molecules with different weights [54].

In a study conducted by Kidambi et al., GOM is characterized as a large-area nanoporous atomically thin membrane. It was obtained by chemical vapor deposition (CVD) where graphene was transferred onto polycarbonate track-etched supports and then etched with oxygen plasma to produce size-selective pores smaller than 1 nm [55]. What is important for blood-contacting materials, graphene nanocoating shows good hemocompatibility with no hemolytic effect on human erythrocytes after 1h incubation [53].

### 8.2. Carbon Nanotubes Modification Possibilities

Carbon nanotubes (CNTs) have attracted considerable attention because of their exceptional electrical conductivity, high mechanical strength and anti-biofouling properties. Single-walled carbon nanotubes (SWCNTs) and multi-walled carbon nanotubes (MWCNTs) differ in size and cytotoxicity, but both show ease in the chemical functionalization, which is found to further influence the properties and broaden their application.

**Figure 6:**
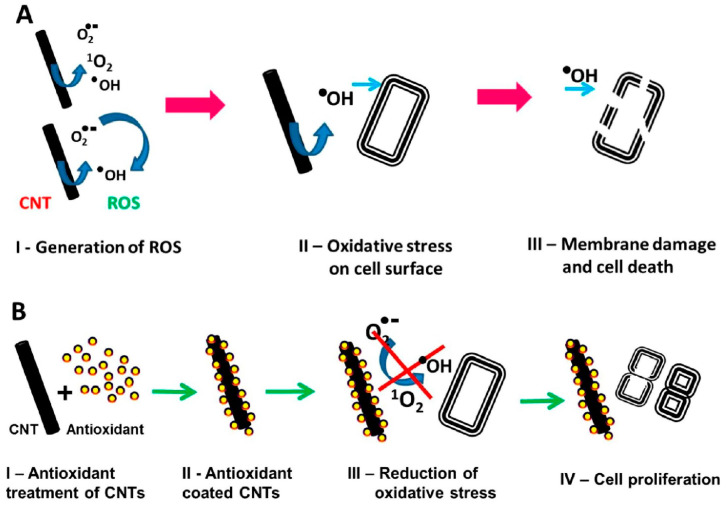
Schematic representation of oxidative stress-meditated cell death and its amelioration by antioxidant treatment. (**A**) ROS-meditated bacteria cell death (**B**) Protection of bacteria cells against oxidative stress by antioxidants. Reprinted with permission from Rajavel, K.; Gomathi, R.; Manian, S.; Rajendra Kumar, R.T. In Vitro Bacterial Cytotoxicity of CNTs: Reactive Oxygen Species Mediate Cell Damage Edges over Direct Physical Puncturing. Langmuir 2014, 30, 592–601 [56]. Copyright 2014 American Chemical Society.

In comparison with graphene, CNT cytotoxicity mechanisms do not involve a physical puncture of the membrane. They mainly act by generating ROS (singlet oxygen, superoxide anions (O2^•−^), and hydroxyl radicals (^•^OH)) through a series of photochemical reactions in aqueous solutions (Figure 6). The peroxidation of lipids in the cell membrane results in the efflux of contents and the eventual death of the cell. However, this seems to only work under UV light and not when cells are incubated in the dark [56].

The use of CNTs in membrane technology began as an additive to improve the mechanical strength of base materials, but because in many studies, they show excellent proprieties in reducing bacterial growth, increasing water flux, and removing heavy metal ions from the water they become popular in membranes used for water treatment and desalination. For example, the PSf-CNT membrane shows retention of Pb^+^ and Hg^+^ superior to that of pristine PSf [57,58].

Membranes with CNTs are manufactured in two ways: as mixed matrix membranes, where CNTs are dispersed in polymer solution forming a loosely connected CNT-network or as membranes where vertically aligned CNTs create cylindrical pores and fluid is forced to pass through nanotube [57].

Kang et al. reported that the diameter of CNT used in membrane production affects antibacterial properties with SWCNT membranes being more cytotoxic than MWCNT membranes [59]. PES membranes with CNT functionalized by a non-covalent bonding with sodium lignosulfonate (SLS) show increased surface hydrophilicity and lower protein adsorption. CNT can be also used for changing the pore size and morphology and thus controlling the size of removed molecules [60].

The antibacterial application of CNT in medical devices is limited by suspected blood-cell toxicity and CNT aggregation in the polymer matrix. One of the offered solutions is to incorporate PEG grafting in the form of TPU-g-PEG/CNT nanofibers. During tests, they demonstrate high hemocompatibility with lower hemolysis ratios and suppressed adhesion of red blood cells onto the surface without a reduction in antibacterial properties [61].

Abidin et al. also investigated the effects of MWCNTs on the biocompatibility and safety of membranes dedicated to HD treatment. Their idea was to obtain PES hemodialysis membrane embedded with PCA-gMWCNTs which demonstrated an enhanced ratio of oxygen-rich groups. The modified nanocomposite membrane showed improved dispersion stability and was considerably more biocompatible and exhibited lower complement activation and protein adsorption compared to the pristine PES membrane. In addition, there was no leaching detected during filtration [62].

### 8.3. Silica Nanoparticles Modification Possibilities

Silica nanoparticles (SNPs) are a major topic in nanoparticle research with an emphasis on biomedical applications like bio-imaging [63] and drug delivery technologies [64,65]. This group of particles includes core-shell silica nanoparticles, nonporous SNPs, hollow mesoporous silica nanoparticles (HMSN), and mesoporous silica nanoparticles (MSN) with pores size diameter between 2 and 50 nm [66]. They are characterized by chemical and physical stability, high biocompatibility and large surface area. Similarly to CNT, by further modification of MSN, we can affect particle properties such as diameter, shape, porosity, and both core and surface features, which subsequently leads to obtaining particles with customized properties [67].

There are a few ways to incorporate silica nanoparticles into antimicrobial strategies, but they all assume the use of SNPs as a vector for releasing antibacterial substances like drugs or nitric oxide [65]. In membrane technology, they can be applied as antimicrobial coatings.

Polymeric membranes with nanosilica additives show increased hydrophilicity [68]. Adding silica results in changes in surface properties indicated by a lower contact angle. One of the suspected reasons behind it may be a homogenous dispersion of nanoparticles, but in this particular case, silica particles are providing more functional groups to the membrane surface which are mainly hydrophilic [69]. Higher hydrophilicity of the membrane in the presence of MSN is also translated into improved anti-fouling surface properties [68].

The porous structure of MSN particles provides an additional passageway for water molecules leading to a significant increase in the water flux. Membrane morphology is also changed by creating more free space by disruption of polymer chain packing and loose interactions between MSN and polymer. Likewise, as more MSN is added, salt rejection rates of membranes decrease [68].

What is important is that MSNs show low systemic toxicity due to natural mechanisms of degradation in the body. Toxicity is dose-dependent, but the LD50 is 1000 mg/kg which exceeds several times the doses used therapeutically (1 to 50 mg/kg) [67]. MSN biocompatibility has been shown in tests with a wide range of cancerous and noncancerous cell lines, but its hemocompatibility can be further enhanced by pegylation and by reducing the interactions between negatively charged silica and positively charged groups in red blood cell membranes [65].

In Table 2 we summarized the antimicrobial properties of described nanoparticles. All three materials have the potential to prevent biofilm formation, but further research is needed to determine their long-term effects, potential cytotoxicity, and effectiveness in real-world medical settings. Both RGO and GO have a unique surface chemistry that enables interactions with bacterial cells, but RGO’s improved electrical conductivity might offer additional antimicrobial effects. Furthermore, CNTs disrupt bacterial cells in a mechanism different from GO’s and RGO’s interactions. In conclusion, each of these carbon-based nanomaterials has distinct advantages and challenges in terms of their antimicrobial properties. The choice of material would depend on factors such as the specific application, material synthesis, toxicity considerations, and the desired mechanism for preventing biofilm formation on membranes used in blood purification therapies like CRRT.

## 9. Conclusions

Continuous renal replacement therapy is a method of extracorporeal blood purification that uses membrane processes such as diffusion and ultrafiltration to remove uremic toxins. Filtration efficiency decreases with time due to the accumulation of plasma proteins, platelets and EPS produced by sedentary colonies of bacteria on the membrane surface. Available studies conducted on marine filters show that biofilm is formed only after several hours. However, there are no similar studies on hemofilters used in CRRT, most likely due to difficulties in obtaining the material for the tests. Currently, the problem of biofilm formation on medical device surfaces is noticed by many scientists. Among the solutions presented in the article, nanoadditives like CNT, graphene, and silica, seem to be the most promising in biofilm mitigation.

## Figures and Tables

**Table 1:** The classic division of uremic toxins is based on the physicochemical properties of molecules. Low-weight molecules have molecular mass lower than 500 Da, middle molecules are 0.5–15 kDa and high molecular weight is higher than 15 kDa which includes protein-bound toxins. Currently, it is postulated to change this division into a more dynamic one.

Name	Size	Blood/Serum Concentration
**Low molecular weight**		
urea	60 Da	15–40 mg/dL
creatinine	113 Da	depends on the patient sex and age, 0.6–1.3 mg/dL
uric acid	168 Da	3–7 mg/dL
**Middle molecular weight**		
Parathyroid hormone	9.5 kDa	15–65 pg/mL
Beta-2-microglobulin	11.8 Da	<1.8 mg/L
myoglobin	18 kDa	<70–110 µg/L
Il-6	23.7 kDa	<1.8 pg/mL

**Table 2:** Comparison of graphene, CNTs and SNPs as antimicrobial modifiers.

Properties	Graphene	CNTs	SNPs
antibacterial mechanism	physical damage, ROS independent oxidative stress, wrapping and trapping	oxidative stress, metabolism disruption	vector for releasing antibacterial substances
membrane modification	coating, graphene oxide-based membranes, mixed matrix membrane	mixed matrix membrane, vertically aligned CNTs	coating, mixed matrix membrane
biocompatibility	high	high	high
surface hydrophilicity	increased	increased	increased
tested in HD membranes	no	no	no

## Abbreviations

AKI	acute kidney injury
APTT	activated partial thromboplastin time
CNTs	carbon nanotubes
CRRT	continuous renal replacement therapy
CVD	chemical vapor deposition
DIC	disseminated intravascular coagulation
EPS	extracellular polymeric substance
EVOH	ethylene vinyl alcohol
FCM	flow cytometry
GO	graphene oxide
GOM	graphene oxide-based membranes
HD	hemodialysis
HMSN	hollow mesoporous silica nanoparticles
LBL	layer by layer
MF	microfiltration
MMM	mixed matrix membrane
MSCRAMMs	microbial surface components recognizing adhesive matrix molecules
MSN	mesoporous silica nanoparticles
MWCNTs	multi-walled carbon nanotubes
PAN	polyacrylonitrile
PES	polyethersulfone
PMA	phosphomolybdic acid
PMMA	polymethylmethacrylate
PSf	polysulfone
PTFE	polytetrafluoroethylene
RGO	reduced graphene oxide
ROS	reactive oxygen species
SEM	scanning electron microscopy
SEM-RR	scanning electron microscopy-ruthenium red
SIMS	secondary ion mass spectrometry
SLS	sodium lignosulfonate
SNPs	silica nanoparticles
SWCNTs	single-walled carbon nanotubes
ToF-SIMS	time-of-flight SIMS
UF	ultrafiltration
UFH	unfractionated heparin
VLA-5	very late antigen 5
VPSEM	variable pressure scanning electron microscopy
XPS	X-ray photoelectron spectroscopy
